# The X-ray Correlation Spectroscopy instrument at the Linac Coherent Light Source

**DOI:** 10.1107/S1600577515004397

**Published:** 2015-04-14

**Authors:** Roberto Alonso-Mori, Chiara Caronna, Matthieu Chollet, Robin Curtis, Daniel S. Damiani, Jim Defever, Yiping Feng, Daniel L. Flath, James M. Glownia, Sooheyong Lee, Henrik T. Lemke, Silke Nelson, Eric Bong, Marcin Sikorski, Sanghoon Song, Venkat Srinivasan, Daniel Stefanescu, Diling Zhu, Aymeric Robert

**Affiliations:** a Linac Coherent Light Source, SLAC National Accelerator Laboratory, 2575 Sand Hill Road, Menlo Park, CA 94025, USA; b Hasylab at DESY, Notkestrasse 85, D-22607 Hamburg, Germany

**Keywords:** FEL, hard X-ray, XPCS, coherent scattering

## Abstract

The X-ray Correlation Spectroscopy instrument is dedicated to the study of dynamics in condensed matter systems using the unique coherence properties of free-electron lasers. It covers a photon energy range of 4–25 keV. The intrinsic temporal characteristics of the Linac Coherent Light Source, in particular the 120 Hz repetition rate, allow for the investigation of slow dynamics (milli­seconds) by means of X-ray photon correlation spectroscopy. Double-pulse schemes could probe dynamics on the picosecond timescale. A description of the instrument capabilities and recent achievements is presented.

## Abstract 2

A description of the X-ray Correlation Spectroscopy instrument at the Linac Coherent Light Source is presented. Recent highlights illustrate the coherence properties of the source as well as some recent dynamics measurements and future directions.

## 1. Introduction   

The Linac Coherent Light Source (LCLS), a US Department of Energy Office of Science user facility operated by Stanford University, achieved first light in 2009 (Emma *et al.*, 2010 ▶). The X-ray Correlation Spectroscopy (XCS) instrument began operations in 2011; located in the Far Experimental Hall (FEH), XCS was the fifth LCLS instrument to become operational. In contrast with synchrotron storage ring sources, which provide high-brilliance beam with partial coherence (Grübel *et al.*, 2008 ▶), LCLS provides pulsed transversely coherent hard X-rays with unprecedented flux and short pulse duration.

These characteristics enable the investigation of dynamics in condensed matter by measuring time-resolved coherent scattering patterns (*i.e.* time-resolved speckles). This can be achieved by means of X-ray Photon Correlation Spectroscopy (XPCS) (Grübel *et al.*, 2008 ▶), where the temporal evolution of speckle patterns can be quantified by calculating intensity autocorrelation functions and extracting typical relaxation times for specific length scales (Robert, 2007 ▶). The LCLS repetition rate, 120 Hz, limits the fastest measurable time scales to several tens of milliseconds (Stephenson *et al.*, 2009 ▶).

More elaborate schemes, requiring the measurement of the sum of two speckle patterns originating from a sequence of two X-ray pulses separated in time (Gutt *et al.*, 2009 ▶), allow the measurement of much faster dynamics. In that case the accessible time scales are limited by the ability to generate two pulses separated in time by 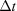 and can potentially reach the femtosecond regime.

The XCS instrument was designed to provide an optimum platform for taking advantage of the unique coherence properties of the LCLS (*e.g.* the possibility to access very large sample–detector distances) while still accommodating experiments using different X-ray techniques (small-angle X-ray scattering, diffraction, X-ray spectroscopy and imaging) (Robert *et al.*, 2013 ▶). The addition of an ultrafast laser system will not only allow ‘standard’ optical pump/X-ray probe experiments but will open new opportunities to use coherent scattering techniques in combination with ultrafast optical excitation.

## 2. Instrument overview   

The XCS instrument operates in the hard X-ray regime (above 4 keV). A set of silicon mirrors (coated with silicon carbide), with an incidence angle of 1.32 mrad, are located in the front-end enclosure of the Near Experimental Hall (NEH), which feeds the hard X-ray beam to all LCLS hard X-ray hutches. These limit the maximum photon energy that can be delivered to the LCLS hard X-ray hutches to 25 keV.

**Figure 1:**
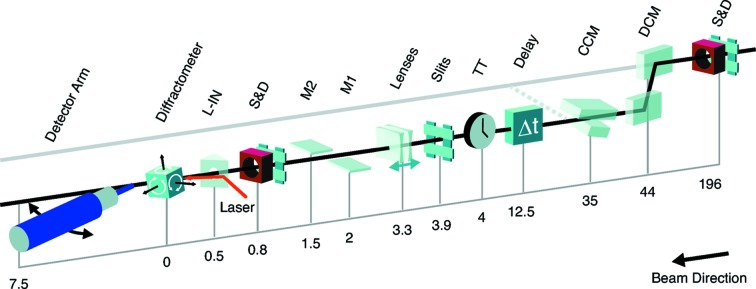
Overview of the XCS instrument layout. Distances are indicated in meters from the center of the diffractometer. S&D: slits and non-destructive intensity diagnostics; DCM: large-offset double-crystal monochromator; CCM: channel-cut monochromator; TT: time-tool measuring the arrival time of the optical laser with reference to the X-rays; M1/M2: silicon mirrors that can be used to deflect the beam in the vertical direction and can also provide harmonic rejection; L-IN: laser in-coupling for the optical laser. Components located downstream of the dashed line can be translated into the main LCLS line and allow the XCS instrument to take advantage of the full power and properties of the fundamental. The sample at the XCS instrument is located approximately 420 m from the source.

The first components of the XCS instrument are located in the X-ray Transport Tunnel, a 200 m-long tunnel connecting the Near and Far Experimental Halls. These include slits and diagnostics located 196 m upstream from the sample, as indicated in Fig. 1 ▶.

In the following, we describe the specifications of key components of the XCS instrument.

*Monochromators.* The XCS instrument has two distinct configurations. It can operate in the LCLS main line and take full advantage of the first-harmonic properties of LCLS and its various operation modes (SASE, two-color, *etc.*). To operate in this ‘pink beam’ configuration, all components located downstream of the dashed line in Fig. 1 ▶ are translated to the LCLS main line. For experiments that require a monochromatic beam [*i.e.* a larger longitudinal coherence length 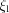 = 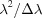 = 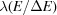] but do not intend to scan broadly the incident energy, a custom-built (JJ X-ray, Denmark) large-offset Double-Crystal Monochromator (DCM) (Zhu *et al.*, 2014 ▶) is located 44 m upstream of the sample. In this configuration all components located downstream of the dashed line are translated 600 mm horizontally as displayed in Fig. 1 ▶. The DCM operates with Si(111) crystals and provides a monochromaticity of 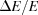 = 1.4 × 10^−4^ at 8 keV. If more monochromaticity is required, a Si(511) Channel-Cut Monochromator (CCM) (Narayanan *et al.*, 2008 ▶) operating in the vertical scattering geometry provides an energy resolution of 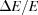 = 8.9 × 10^−6^. Future plans include an upgrade of the DCM crystals to 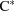
(111), which not only provides better energy resolution (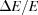 = 5.3 × 10^−5^) but most importantly allows multiplexing with another instrument located downstream in the FEH (Feng *et al.*, 2013 ▶, 2015 ▶; Zhu *et al.*, 2014 ▶). A detailed discussion concerning the effect of the monochromaticity on the intensity and longitudinal coherence properties of a FEL SASE beam is provided by Lee *et al.* (2012) ▶.

*X-ray focusing lenses.* The unfocused beam at XCS is typically 0.75 mm × 0.75 mm FWHM in size. Beryllium compound refractive lenses (RWTH, Aachen, Germany) allow focusing of the beam, and provide beam size control in one or two dimensions (Snigirev *et al.*, 1996 ▶). The lenses are located 3.3 m upstream of the sample and can be adjusted ±150 mm along the incident beam axis. The minimum spot size was measured to be ∼2 µm × 2 µm (limited by the finite SASE bandwidth and imperfections in the optics). A one-dimensional focusing option allows delivery of a line focus for elongated samples or for applications such as grazing-incidence experiments.

*Pulse picker.* A fast shutter is available for selecting single X-ray pulses from the LCLS pulse train on demand, as well as for reducing the repetition rate. It consists of a channel which is rotated back and forth in order to create a brief opening time. It can be used to create arbitrary pulse train time patterns provided that the pulse train structure has an average rate of equal to or less than 10 Hz.

*Mirrors.* Two silicon mirrors located 1.5 and 2 m upstream of the sample allow delivery of the beam with a vertical grazing angle (as, for example, is required for liquid surfaces). These can also reduce the third-harmonic content of the LCLS beam by operating above the critical angle for these energies.

*Diffractometer.* A custom-built horizontal geometry four-circle diffractometer (Huber, Germany) is available and enables precise orientation of samples or sample environments such as vacuum chambers, gas/liquid injectors, *etc*. Its sphere of confusion is better than 20 µm. It can be used in conjunction with the auxiliary 2θ arm (FMB Oxford, UK) providing large sample–detector distances (4 and 7.5 m) and covering scattering angles up to 55° in the horizontal plane.

*Additional diagnostics.* The SASE process induces pulse-to-pulse fluctuations of the beam properties, such as pulse energy, duration, spatial profile, wavefront, temporal profile and spectral content. *In situ* pulse property monitoring is thus crucial for data interpretation. Multiple intensity monitors are installed at various locations along the instrument for pulse-to-pulse intensity normalization (Feng *et al.*, 2011 ▶). The spatial profile of the beam can also be measured at various locations along the instrument using scintillating screens with high-resolution camera–lens combinations.

*Detectors.* Several X-ray detectors are available and integrated with the XCS data acquisition system. These have various characteristics (pixel size, number of pixel, noise, frame rate and dynamic range) which are evaluated in order to identify the most suitable detector for specific experimental needs. Coherent X-ray experiments, for example, typically require small pixel size, very low noise, moderate dynamic range and a large number of pixels. This can be achieved with the 20 µm pixel size direct illumination Princeton CDD, but at a very low frame rate. A new detector is currently being developed at LCLS with 55 µm pixels and low noise, but running at the 120 Hz full repetition rate (Dragone *et al.*, 2014 ▶). For more information about the LCLS detectors, see Blaj *et al.* (2015) ▶.

*Split and delay.* The XCS instrument has space allocated for instrumentation to generate double-pulse X-ray patterns with a controlled 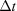 typically below 1 ns. A split and delay prototype built by DESY (Hamburg, Germany) is currently installed and its performances are described elsewhere (Roseker *et al.*, 2009 ▶, 2012 ▶). Other prototypes offering different beam properties are being evaluated or tested.

Ultrafast laser capabilities will be added to the XCS instrument in 2015. These include the construction of a dedicated laser hutch in close proximity to XCS, a standard ultrafast laser system, and timing diagnostics; the specifications of each are listed below:

*Optical laser system.* Core laser systems at the LCLS consist of an ultrashort-pulse Ti:sapphire oscillator synchronized to the FEL seeding a commercially available chirped pulse amplifier producing 4 mJ at 40 fs. An additional four-pass amplifier, developed in-house, can boost the pulse energy to over 30 mJ. Wavelength conversion can cover a broad spectral range from 200 nm to 150 µm (∼1500 to 2 THz). A more thorough description of the optical laser capabilities at LCLS can be found by Minitti *et al.* (2015 ▶).

*Timing diagnostics.* Typical phase locking between the accelerator and the laser system only holds the temporal jitter between the two sources to about 200 fs FWHM. In order to take full advantage of the short pulses and reach pulse-length-limited time resolution, diagnostics to measure the relative arrival time between laser and X-ray pulses have been developed. These are based on the X-ray induced change in refractive index of a thin target probed by a chirped broadband white-light continuum pulse derived from the optical laser. The optical light transmission change is resolved by an optical spectrometer for each pulse. Typical target materials are silicon nitride (Si_3_N_4_) or Ce:YAG crystals of different thicknesses to accommodate different beam conditions (Lemke *et al.*, 2013 ▶).

A summary of the XCS instrument parameters is given in Table 1 ▶.

## 3. Highlights   

**Figure 2:**
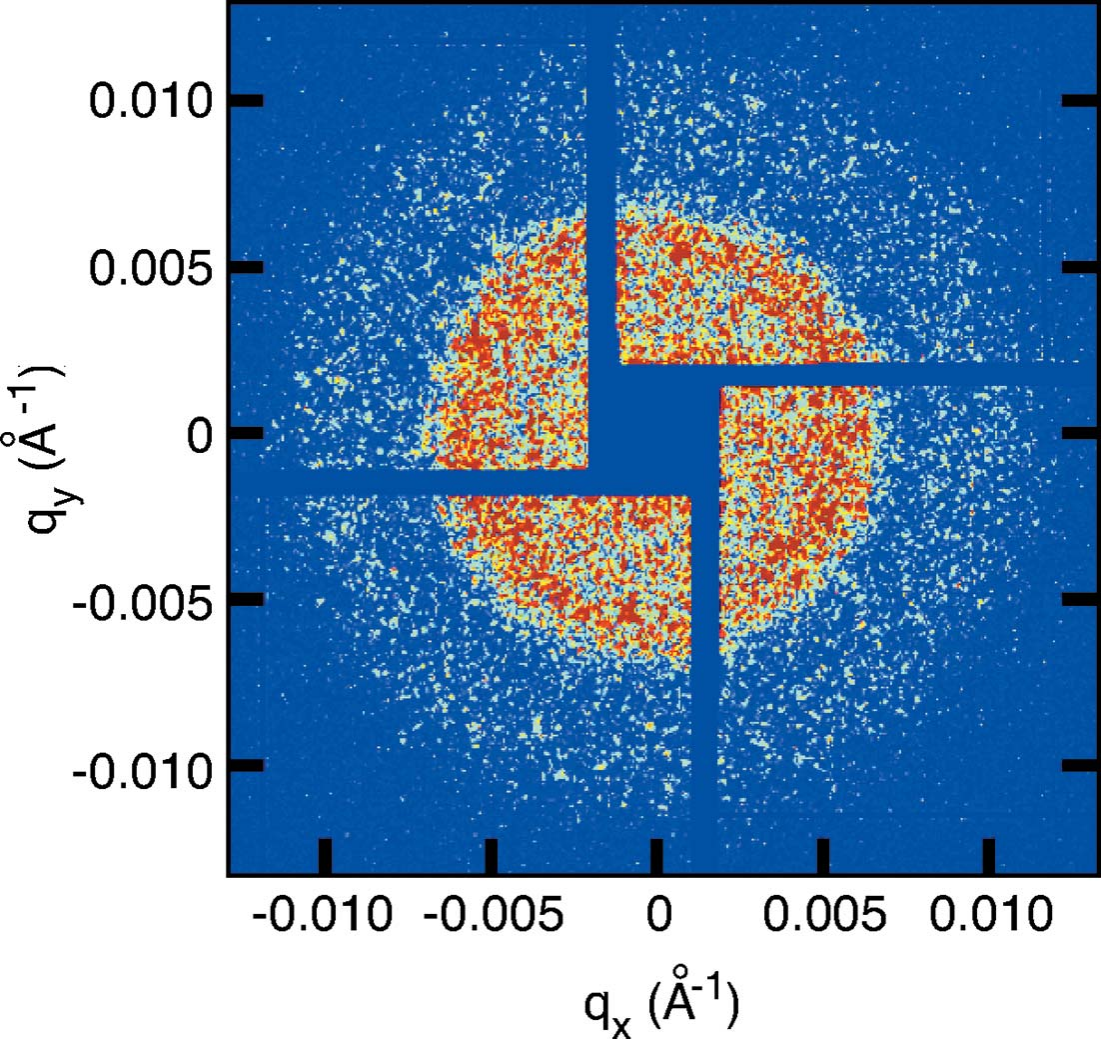
Single-shot speckle pattern measured at 8.3 keV from 150 nm colloidal spheres. The dark blue areas are gaps between the CSPAD tiled sensors. The central aperture allows the transmitted beam to pass through, and therefore does not require a beamstop.

The XCS instrument focuses on measuring time-resolved coherent scattering patterns from condensed matter systems from which typical relaxation rates are deduced. These measurements take full advantage of the transverse coherence properties of the LCLS beam. Fig. 2 ▶ displays a typical single-shot speckle pattern from a static sample consisting of dried silica colloidal 150 nm diameter spheres (Kisker GmbH) with 8.3 keV X-rays. The detector is a CSPAD (Blaj *et al.*, 2015 ▶) located 7.5 m downstream of the sample. The concentric excess scattering signals observed at *Q* ≃ 0.005 Å^−1^ and *Q* ≃ 0.01 Å^−1^ are typical small-angle scattering form-factor features related to the size and shape of the colloidal particles. As observed, the speckles (coherent scattering pattern appearing as the grainy features decorating the structure rings) are well developed.

**Figure 3:**
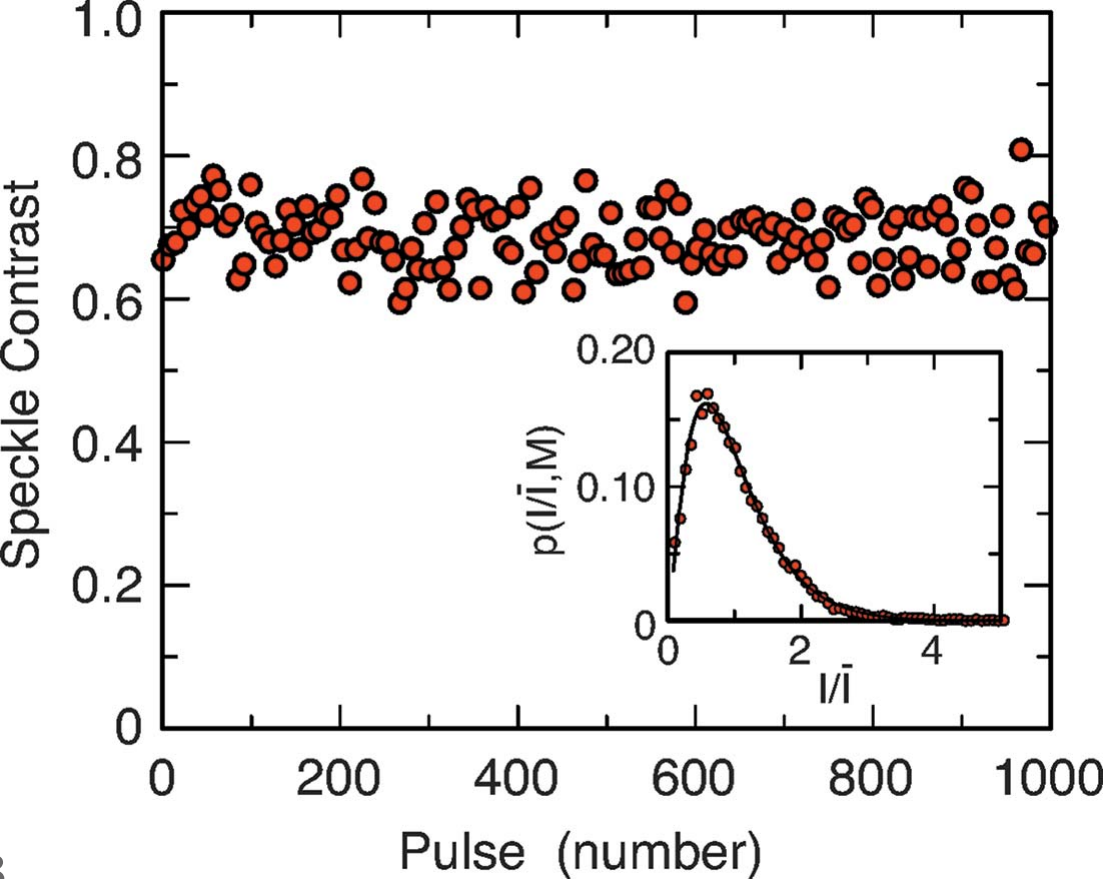
Single-shot speckle contrast measured at *Q* = 0.0067 Å^−1^ for various consecutive shots. Inset: probability density function of intensity within part of the speckle pattern corresponding to a wavevector *Q* = 0.0067 Å^−1^. The solid line represents the gamma distribution with number of modes *M* = 2.75 and average count rate 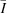 ≃ 5.1 photons.

In order to characterize the transverse coherence of the beam, a series of speckle patterns are measured with and without the XCS monochromator, in the SASE operation mode, and analyzed in terms of photon statistics to determine their contrast, *i.e.* a measure of the transverse coherence in the small-angle regime (Gutt *et al.*, 2012 ▶; Hruszkewycz *et al.*, 2012 ▶; Lee *et al.*, 2013 ▶; Lehmkühler *et al.*, 2014 ▶). The intensities from a narrow iso-*Q* area consisting of an annulus centered at *Q* = 0 with a radius *Q* are histogrammed as displayed in Fig. 3 ▶ for *Q* = 0.0067 Å^−1^. These are then modeled using the negative-binomial distribution function (Mandel, 1959 ▶; Goodman, 2007 ▶)
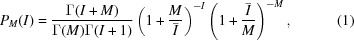
where *I* is the number of photons, 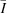 is the mean number of photons in that area and *M* is the number of modes. *M* is related to the contrast of the speckle pattern *C* = 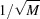. An example of the result of such analysis is displayed in Fig. 3 ▶ (inset). For that specific shot the fit to the experimental data yields a mean number 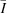 ≃ 5.1 photons, mode number 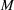 = 2.75 corresponding to a contrast 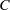 = 0.6. The fit reproduces well the experimentally measured single-shot intensity distribution.

Because the mean number of photons is large one can also use a simpler formulation of the speckle contrast *C* = 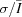, where σ is the standard deviation of the measured intensities. This analysis was performed on successive shots at the same *Q* = 0.0067 Å^−1^. The results are displayed in Fig. 3 ▶. The observed contrast fluctuates between 0.6 and 0.8 with a mean contrast of 0.69. The shot-to-shot fluctuations of the contrast originate from the fine structures in the energy spectrum of the SASE beam, as simulated and described by Lee *et al.* (2012) ▶.

The large degree of coherence of the LCLS beam makes it a suitable source to perform XPCS, measuring time-resolved speckle patterns from a sample. As the sample presents some dynamics, speckle patterns fluctuate in time. By means of time-autocorrelation functions calculated from the speckle patterns, information on the characteristic relaxation times can be obtained (Robert, 2007 ▶), a measure of the normalized intermediate scattering function of the system. In some cases the sample can also undergo non-equilibrium dynamical phenomena, referred to as aging (Robert *et al.*, 2006 ▶), from which clear signatures can be observed in a time-dependent analysis of the degree of correlation of speckle patterns. This was recently investigated at XCS by Carnis *et al.* (2014) ▶, where the relaxation and aging dynamics of thin polymer films were investigated. One should also note that with a FEL the spatial position of the beam jitters shot-to-shot. Because of this effect the contrast β, usually referred to in second-order correlation functions (Robert, 2007 ▶), will be smaller than the single-shot speckle contrast, as discussed by Carnis *et al.* (2014 ▶).

Time-resolved speckle spectroscopy at an FEL is intrinsically limited by the repetition rate of the source, as it relies on the time-correlation of two recorded coherently scattered patterns originating from two pulses separated in time. For the LCLS, the fastest dynamics that can be reached is of the order of 8.3 ms (*i.e.* 120 Hz repetition rate). A natural way of accessing faster timescales is to increase the repetition rate of the source. This would require a completely different accelerator technology such as the one planned to be used at the European XFEL (Grübel *et al.*, 2007 ▶), where series of X-ray bunches separated by a minimum of 220 ns will be generated.

**Figure 4:**
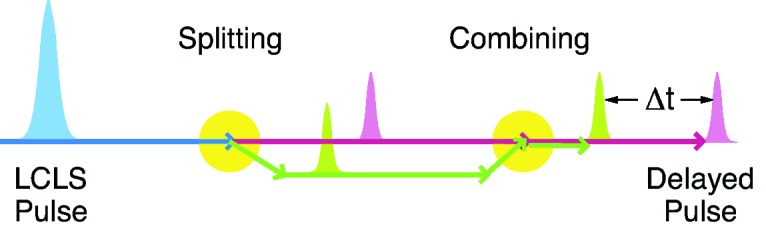
Schematic of the double-pulse scheme. A single pulse is split into two sub-pulses; one is delayed relative to the other by an increase in its pathlength. The sub-pulses, separated in time by 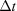, are then redirected on a common trajectory to the sample. A typical pathlength difference of 1 mm corresponds to a time delay of 3 ps.

To reach even faster timescales down to the picosecond regime and below, other possibilities involve generating two sub-pulses with a time separation 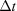 between tens of femtoseconds up to a nanosecond as illustrated in Fig. 4 ▶. Each sub-pulse generates a speckle pattern. Current X-ray detectors are, however, only capable of measuring the sum of the scattered signal of both sub-pulses. If dynamics occurs on timescales of 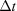, the summed speckle patterns will present a decay of contrast, as compared with the contrast of a single speckle pattern. By adjusting the time delay 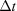 between the two sub-pulses, one can extract the time evolution of the summed speckle pattern contrast, from which the normalized intermediate scattering function is obtained (Gutt *et al.*, 2009 ▶), and therefore gain information about the underlying dynamics of the system.

Recent results from the commissioning of a split and delay prototype (Roseker *et al.*, 2012 ▶) based on eight perfect Bragg crystals [including two thin crystals that can act as a beam splitter/recombiner (Roseker *et al.*, 2009 ▶)] have shown the possibility to reach time delays of the order of a nanosecond.

Other technical developments involving split and delayed beams have also been investigated. This is particularly the case with a single-shot split and multiple-delay system, offering the possibility to probe ultrafast dynamics by means of X-ray pump/X-ray probe experiments, as recently demonstrated by David *et al.* (2015) ▶.

## 4. Conclusion   

The XCS instrument takes full advantage of the large number of transversely coherent photons per pulse at the LCLS. XCS is a versatile tool for performing time-resolved coherent scattering experiments in the hard X-ray regime from which fundamental dynamics in condensed matter ordered and disordered systems can be explored. This can be achieved by means of XPCS for slow dynamics and will further be extended to ultrafast timescales by double-pulse experiments. More details about the XCS instrument can be found on the following website: http://lcls.slac.stanford.edu/xcs. 

## 5. Facility access   

LCLS instruments are open to academia, industry, government agencies and research institutes worldwide for scientific investigations. There are two calls for proposals per year and an external peer-review committee evaluates proposals based on scientific merit and instrument suitability. Access is without charge for users who intend to publish their results. Prospective users are encouraged to contact instrument staff members to learn more about the science and capabilities of the facility, and opportunities for collaboration.

## Figures and Tables

**Table 1:** X-ray parameters and capabilities of the XCS instrument

Instrument name	XCS
Mirrors, incidence angle	2 SiC on Si, 1.32mrad
Monochromaticity ( 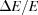 )†	1 10^3^ (SASE), 2 10^4^ (seeding)
Energy range (keV)	4 to 11 (fundamental)
Unfocused beam size (m)	750 at 8.3keV
Focused beam size (m)	2750
Focusing optics	Be lenses, 1D and 2D focusing
Flux (photons pulse^1^)	1 10^12^ (fundamental)‡
Pulse length (fs)	5200
Repetition rate (Hz)	120, 60, 30, 10, 5, 1, on demand
Optical laser pulse energy (mJ)§	20 (800nm), 45 (400nm), 1 (266nm)
Optical laser pulse length (fs)§	10150
Standard detectors	CSPAD, CSPAD-140k, ePix
	Princeton
Sample environment	Huber horizontal four-circle diffractometer, general purpose vacuum, liquid jet, He enclosure, Oxford LN_2_ cryojet down to 100K
2 arm capabilities	Large sampledetector distance arm (4 and 7.5m) covering 0 2 55

† Typical single-shot value.

‡ Excluding beamline and instrument transmission.

§ To be installed in 2015.
